# Multimodal Therapy in Multidrug-Resistant Prostatitis: A Case Report and Review of the Literature

**DOI:** 10.7759/cureus.74025

**Published:** 2024-11-19

**Authors:** Elise Renaud, Rajeev P Nagassar, Satyendra Persaud

**Affiliations:** 1 Surgery, Sangre Grande Hospital, Sangre Grande, TTO; 2 Microbiology, Sangre Grande Hospital, Sangre Grande, TTO; 3 Urology, Division of Clinical Surgical Sciences, University of the West Indies, Saint Augustine, TTO

**Keywords:** antimicrobial resistance, chronic bacterial prostatitis, injection therapy of prostatitis, intra-prostatic antibiotics, intra-prostatic injections, multidrug-resistant prostatitis, recurrent resistant prostatitis

## Abstract

Chronic bacterial prostatitis is generally difficult to treat. It may involve multiple courses of antibiotics and may be difficult to eradicate with high rates of recurrence. We present the case of a 33-year-old male patient with a previous history of renal tract calculi, stent insertions, and perinephric abscess with percutaneous drainage, which then led to a right nephrectomy. He eventually developed recurrent urinary tract infections with the prostate as the source. This patient had at least two prior, prolonged courses of intravenous antibiotics, one consisted of two weeks and the other of six weeks of intravenous meropenem at different times. His last episode of prostatitis was managed with intraprostatic injections of lidocaine and a mixture of amikacin and meropenem, given at once-a-week intervals for four weeks along with simultaneous thrice-daily meropenem during this period.

## Introduction

Chronic bacterial prostatitis (CBP) is characterized by chronic or recurrent urogenital symptoms with evidence of prostatic infections caused by the same organism lasting at least three months [1]. It is frequently associated with chronic prostate gland pain. We aim to describe another option for the treatment of CBP resistant to repeated courses of antibiotics. The increase in antimicrobial resistance can lead to the recurrence of CBP after oral therapy. Mayersak declared chronic prostate gland pain as the most common and difficult condition of the urological profession to manage [2]. Complications, after intra-prostatic injections, were only minor, with hematospermia, gross hematuria, and mild pain. Mayersak defined a 90% cure rate by the absence of symptoms after treatment with transperineal aminoglycoside injection for "hard-core" prostatitis [2].

Overall, 4-14% of men presenting with prostatitis symptoms may have CBP [3,4]. Within the past 40 years, as stated in the literature, after multiple attempts of antibiotic treatment and repeated failures, symptoms reappear when antibiotics are discontinued. Our aim was to increase the contribution to the international literature on the use of intra-prostatic and intravenous injections with carbapenems against multi-drug resistant organisms. Zhang et al. concluded that this was a safe and effective way with a 92.5% success rate, with no relapse in 28 out of 40 follow-up cases [5]. It delivers a safe and practical approach, thereby increasing understanding, providing effective and alternative treatment options, better symptom scores, decreasing pain and irritative and obstructive symptoms, and contributing to a better quality of life.

Total prostatectomy is also a consideration. Shafik suggested that total prostatectomy would provide a definitive cure; however, there is a high risk of urinary incontinence and impotence postoperatively, and trans-urethral resection of the prostate (TURP) provides only a 33% cure rate [6]. We could not find any cases in the literature on the effective treatment of resistant recurrent prostatitis in the Caribbean. There is a lack of evidence of empirical versus standardized care. Zvara et al. noted that there are still inconsistencies in study design, management modalities, outcome measures, methods and thus results in the literature [7]. In most cases, symptom scores were used to guide management. If the assessment of symptom severity is standardized, management should be too. In agreement, Yamamoto et al. from Japan recommend longer follow-up and more randomized controlled trials (RCTs) [8].

## Case presentation

A 33-year-old male patient presented with a psoas abscess following a left ureteroscopy. This required percutaneous drainage. He subsequently developed a long-segment ureteral stricture and ultimately required a left nephrectomy. He required several hospital admissions including urethral catheterization. Notably, following the nephrectomy, he developed several symptomatic urinary tract infections due to multidrug-resistant *Klebsiella pneumoniae*. CT scan of his abdomen showed no source of infection but subsequent semen cultures confirmed colonization of his prostate with multidrug-resistant *K. pneumoniae*. The bacteria were likely the nidus of infection. He underwent prolonged courses of antibiotics including two weeks of intravenous meropenem followed by six weeks of intravenous meropenem.

Given the failure of intravenous antibiotics, intraprostatic infiltration was carried out. A 25 cm, 23-gauge needle was used to inject a mixture of lidocaine, amikacin, and meropenem transrectally using ultrasound as a guide. Under ultrasound guidance, this was injected throughout the prostate, including the seminal vesicles, at 20 points. This was repeated once weekly for four weeks along with a simultaneous course of intravenous meropenem. Treatment was associated with minor pain and post-procedure hematuria only. After treatment, sterile urine and semen cultures were obtained eight weeks later and the patient has remained asymptomatic for two years. 

The Phoenix™ automated identification and susceptibility testing system (Becton, Dickinson and Company, Franklin Lakes, New Jersey, United States) was used for the identification and antimicrobial susceptibility testing. The Clinical Laboratory Standards Institute (CLSI) M100 guidelines were used for the interpretation of antimicrobial susceptibility data [9].

## Discussion

Intraprostatic injections with simultaneous intravenous meropenem proved to be an effective option. It led to the successful clearance of the patient’s multidrug-resistant recurrent prostatitis. Management of multidrug-resistant recurrent prostatitis or CBP remains challenging. This article presents valuable consideration in particularly difficult cases.

The intraprostatic treatment in our case study was comparable to that by Toth et al., who used a standard endo-cavitary probe, equipped with a needle guide and a 22-gauge spinal needle to deliver 12 ml of the antibiotic cocktail per injection; 3 ml each to the right and left seminal vesicles and to the right and left lobes of the prostate [10]. Each 10 ml volume of the cocktail contained the following antibiotics: gentamicin, 80 mg, clindamycin, 150 mg, metronidazole, 10 mg, moxifloxacin, 3.2 mg, fluconazole, 2 mg, and azithromycin, 50 mg. Methylprednisolone, 50 mg was added to this mixture to activate intracellular dormant reticulate bodies of *Chlamydia trachomatis*. Azithromycin and methylprednisolone were reconstituted in 10 ml of 1% lidocaine each. Follow-up was then at three months, 3-12 months, 12 months or more thereafter. Similarly, Guercini et al., in 2005, tested under ultrasound guidance, an intraprostatic infiltration of a cocktail of antibiotics and steroids [11]. Each patient received dexamethasone 12 mg combined with rifampicin 300 mg and levofloxacin 25 mg. Where *Neisseria gonorrhoeae* was present, levofloxacin was replaced with ceftriaxone 1 g. In the presence of yeasts, fluconazole 6 mg was added, and in the presence of protozoa, metronidazole 15 mg. However, administration was repeated after seven and 14 days, and a complete treatment included three infiltrations, performed on the first, the 10th, and the 20th day. Zhang et al., in 2004, used a sensitivity-guided antimicrobial agent, mixed with lidocaine and dexamethasone, at once weekly intervals, four times, similar to our method of treatment, and evaluated after one month and followed up for six months [5]. Cheng et al. employed an intraprostatic mixture of prednisolone, antibiotics, and lidocaine in 2003 [12]. Plomp et al. utilized 2 g thiamphenicol glycinate injected directly into the prostate [13].

Toth et al. conducted a retrospective analysis of 77 male patients before and after intraprostatic antibiotics and reported a 40-60% improvement in symptom scores [10]. Patients with more than five injections and a short (three-month) follow-up phase showed the most improvement in symptom scores. The inclusion criteria, not clearly stated in all studies examined, were a failure of repeated cycles of antibiotics in the previous 12 months and the severity of the symptoms with a National Institutes of Health-Chronic Prostatitis Symptom Index (NIH-CPSI) ≥ 21 [11]. A 68% cure rate was established, judged by symptom scores and patients' views on overall improvement.

Our case is similar to that of Yamamoto et al., in which intraprostatic antibiotic administrations were followed by follow-ups at three-month intervals for 12 months thereafter [8]. Additionally, Jiminez-Cruz et al. utilized intraprostatic amikacin 500 mg or tobramycin 100 mg, given weekly for two to four weeks then followed up at four, 12, and 24 weeks [14].

Yamamoto et al., in 1996, published a complete cure in 56% of 25 patients who had CBP and were treated with intraprostatic antibiotic administration [8]. Follow-up at three-month intervals for 12 months thereafter using persistently sterile cultures of urine and prostatic secretion were established. In Belgium, Baert and Leonard saw a 71% remission rate amongst 24 patients with CBP treated with direct transperineal infiltration of antibiotics [15]. Unlike Yamamoto et al., we utilized the transrectal route in the current study.

The advantages of guided intraprostatic injections proved effective with minimal side effects and discomfort. It can be done on an outpatient basis, as a concentration of the antibiotics remains in the prostate gland. In a study by Feng, it was shown that most antibacterial agents were not detected in prostatic fluid in patients who still had therapeutic levels in plasma, failing to diffuse from plasma to prostatic fluid [16]. It was emphasized that antibiotics were able to cross the lipoprotein epithelial membrane barrier of the prostate with a high concentration in the gland, thereby giving longer effects. In the experiment by Feng, a 97% cure rate was obtained among 75 patients administered intra-prostatic aztreonam [16]. It appears that a higher concentration of the antimicrobial agent reaches the prostatic tissue compared to that of the serum [6]. In the study by Baert and Leonard, regardless of the time after injection, levels of antibiotics were still high within prostatic fluid, indicating that out of the various management options for refractory bacterial prostatitis, the anal submucosal route seemed more efficacious in reaching the prostate [15]. Furthermore, one study demonstrated that misonidazole (a radiation sensitizer) in the anal submucosa showed a concentration of the drug as high as three times the serum level [6].

Additionally, according to the literature, comparable to this case, there are alternative routes along with the intraprostatic route. Where intramuscular injection and anal submucosal injection (ASI) were employed and compared, the apparent effective rate of the ASI group was 43.3% [15]. There is very little consequence, as mild focal submucosal infiltration of lymphocytes and plasma cells found at seven days after cessation of therapy, disappears by three months after cessation of therapy [17]. Yamamoto et al.'s study yielded up to a 73% success rate in administering oral and intraprostatic antibiotics [18]. Various treatment options are cited including fosfomycin and cycloferon combined with azithromycin [19,20]. Alternatively, surgical removal of infected prostatic tissue, chronic oral antibiotic suppression, and an emerging novel therapy utilizing bacteriophages to target antibiotic-resistant bacteria are suggested [19].

Notably, in our case, we utilized both intravenous and intraprostatic injections. Many alternative routes of antibiotic delivery are described. For example, in the study by Hu et al., in 2002, which included a group of 50 patients, 30 were given amikacin 400 mg via the anal submucosal route daily 10 times, whereas the other 20 cases were given an intramuscular injection for the same amount of time [17]. They were assessed before treatment and at seven and 90 days after treatment. Shafik made use of gentamicin via the anal submucosal route, daily, for 10 days on an outpatient basis, followed by negative cultures by the end of treatment and till two to three years after [6].

## Conclusions

The prostate should be considered the source of infection in patients with recurrent positive cultures from the semen. Intraprostatic therapy may be offered as second-line treatment in patients who fail oral or intravenous therapies. We suggest the transperineal route over the transrectal route. Our case serves as a reminder and a blueprint for treatment. 

## References

[1] Sharp VJ, Takacs EB, Powell CR (2010). Prostatitis: diagnosis and treatment. Am Fam Physician.

[2] Mayersak JS (1998). Transrectal ultrasonography directed intraprostatic injection of gentamycin-xylocaine in the management of the benign painful prostate syndrome. A report of a 5-year clinical study of 75 patients. Int Surg.

[3] Nickel JC, Downey J, Johnston B, Clark J (2001). Predictors of patient response to antibiotic therapy for the chronic prostatitis/chronic pelvic pain syndrome: a prospective multicenter clinical trial. J Urol.

[4] Schneider H, Ludwig M, Hossain HM, Diemer T, Weidner W (2003). The 2001 Giessen Cohort Study on patients with prostatitis syndrome--an evaluation of inflammatory status and search for microorganisms 10 years after a first analysis. Andrologia.

[5] Zhang J, Yang J, He S, Zhang X (2004). Transperineal puncture drug injection in the treatment of chronic prostatitis [Article in Chinese]. Zhonghua Nan Ke Xue.

[6] Shafik A (1992). Perineal versus anal submucosal injection for drug administration in treatment of chronic prostatitis. Urology.

[7] Zvara P, Folsom JB, Plante MK (2004). Minimally invasive therapies for prostatitis. Curr Urol Rep.

[8] Yamamoto M, Hibi H, Satoshi K, Miyake K (1996). Chronic bacterial prostatitis treated with intraprostatic injection of antibiotics. Scand J Urol Nephrol.

[9] (2024). CLSI M100 Performance Standards for Antimicrobial Susceptibility Testing, 34th Edition.

[10] Toth A, Guercini FM, Feldthouse DM, Zhang JC (2018). Injection therapy for chronic prostatitis: a retrospective analysis of 77 cases. Arch Ital Urol Androl.

[11] Guercini F, Pajoncini C, Bard R, Fiorentino F, Bini V, Costantini E, Porena M (2005). Echoguided drug infiltration in chronic prostatitis: results of a multi-centre study. Arch Ital Urol Androl.

[12] Cheng JP, Dai JX, Liu YQ, Hong HW, Zhang XL, Zhang MR, Ye QY (2003). Anatomic observation of the prostate for clinical application of local drug injection through the rectum for chronic prostatitis [Article in Chinese]. Di Yi Jun Yi Da Xue Xue Bao.

[13] Plomp TA, Baert L, Maes RA (1980). Treatment of recurrent chronic bacterial prostatitis by local injection of thiamphenicol into prostate. Urology.

[14] Jiménez-Cruz JF, Tormo FB, Gómez JG (1988). Treatment of chronic prostatitis: intraprostatic antibiotic injections under echography control. J Urol.

[15] Baert L, Leonard A (1988). Chronic bacterial prostatitis: 10 years of experience with local antibiotics. J Urol.

[16] Feng YP (1991). Intraprostatic injection of azactam for chronic prostatitis [Article in Chinese]. Zhonghua Wai Ke Za Zhi.

[17] Hu WL, Zhong SZ, He HX (2002). Treatment of chronic bacterial prostatitis with amikacin through anal submucosal injection. Asian J Androl.

[18] Yamamoto M, Kanai S, Natsume H (1985). The effectiveness of a combination of trimethoprim plus rifampicin and local injection of antibiotics into the prostate in chronic bacterial prostatitis [Article in Japanese]. Hinyokika Kiyo.

[19] Su ZT, Zenilman JM, Sfanos KS, Herati AS (2020). Management of chronic bacterial prostatitis. Curr Urol Rep.

[20] Shperling NV, Vengerovskiĭ AI, Shperling IA, Mironiuk DV, Bezmenova MA, Kalaĭda EV (2011). Treatment of chlamydial infection in chronic prostatitis [Article in Russian]. Urologiia.

